# The proteases HtrA2/Omi and UCH-L1 regulate TNF-induced necroptosis

**DOI:** 10.1186/1478-811X-11-76

**Published:** 2013-10-03

**Authors:** Justyna Sosna, Susann Voigt, Sabine Mathieu, Dieter Kabelitz, Ahmad Trad, Ottmar Janssen, Catherine Meyer-Schwesinger, Stefan Schütze, Dieter Adam

**Affiliations:** 1 Institut für Immunologie, Christian-Albrechts-Universität zu Kiel, Michaelisstr. 5, 24105 Kiel, Germany; 2 Biochemisches Institut, Christian-Albrechts-Universität zu Kiel, Olshausenstr. 40, 24098 Kiel, Germany; 3 Zentrum für Innere Medizin, Nephrologie, Universitätsklinikum Hamburg-Eppendorf, Martinistr. 52, 20246 Hamburg, Germany

**Keywords:** Tumor necrosis factor, Necroptosis, Programmed necrosis, Apoptosis, Proteases, HtrA2/Omi, UCH-L1, Kidney failure

## Abstract

**Background:**

In apoptosis, proteolysis by caspases is *the* primary mechanism for both initiation and execution of programmed cell death (PCD). In contrast, the impact of proteolysis on the regulation and execution of caspase-independent forms of PCD (programmed necrosis, necroptosis) is only marginally understood. Likewise, the identity of the involved proteases has remained largely obscure. Here, we have investigated the impact of proteases in TNF-induced necroptosis.

**Results:**

The serine protease inhibitor TPKC protected from TNF-induced necroptosis in multiple murine and human cells systems whereas inhibitors of metalloproteinases or calpain/cysteine and cathepsin proteases had no effect. A screen for proteins labeled by a fluorescent TPCK derivative in necroptotic cells identified HtrA2/Omi (a serine protease previously implicated in PCD) as a promising candidate. Demonstrating its functional impact, pharmacological inhibition or genetic deletion of HtrA2/Omi protected from TNF-induced necroptosis. Unlike in apoptosis, HtrA2/Omi did not cleave another protease, ubiquitin C-terminal hydrolase (UCH-L1) during TNF-induced necroptosis, but rather induced monoubiquitination indicative for UCH-L1 activation. Correspondingly, pharmacologic or RNA interference-mediated inhibition of UCH-L1 protected from TNF-induced necroptosis. We found that UCH-L1 is a mediator of caspase-independent, non-apoptotic cell death also in diseased kidney podocytes by measuring cleavage of the protein PARP-1, caspase activity, cell death and cell morphology. Indicating a role of TNF in this process, podocytes with stably downregulated UCH-L1 proved resistant to TNF-induced necroptosis.

**Conclusions:**

The proteases HtrA2/Omi and UCH-L1 represent two key components of TNF-induced necroptosis, validating the relevance of proteolysis not only for apoptosis, but also for caspase-independent PCD. Since UCH-L1 clearly contributes to the non-apoptotic death of podocytes, interference with the necroptotic properties of HtrA2/Omi and UCH-L1 may prove beneficial for the treatment of patients, e.g. in kidney failure.

## Background

Cleavage of proteins by caspases is essential for the apoptotic elimination of unwanted or potentially harmful cells and thus for the survival and homeostasis of multicellular organisms [1]. Whereas apoptosis represents the primary route to programmed cell death (PCD) in most physiological settings, non-apoptotic, caspase-independent forms of PCD have been discovered which can act as a backup mechanism to allow cell suicide under conditions where the caspase machinery is inhibited (e.g. in apoptosis-resistant tumor cells or after virus infection) [2,3]. As the main mode of caspase-independent PCD, programmed necrosis (also termed “necroptosis” when mediated by the kinases RIPK1 and RIPK3) has emerged as an important and physiologically relevant response in vital processes, e.g. the elimination of chondrocytes, virus infection, bacterial infection [4] or the homeostasis of T cell populations [5]. Moreover, programmed necrosis has been described to trigger pathophysiological alterations such as neurodegeneration [6], β-cell elimination from pancreatic islets/development of diabetes, loss of hypertrophic cardiomyocytes during heart failure [7], Crohn’s disease [8], acute pancreatitis, ischemic injury and inflammation [4,9,10].

At the molecular level, the signaling pathways of programmed necrosis and necroptosis are still incompletely understood. The best studied model of programmed necrosis, necroptosis mediated by the 55 kDa tumor necrosis factor (TNF) receptor (TNF-R1) depends on the activity of the kinases RIPK1 and RIPK3 and the protein MLKL. These essential core components relay the necroptotic signal to further downstream effectors such as PGAM5L/S and Drp-1, thereby inducing mitochondrial fragmentation [11]. Independently, production of reactive oxygen species, e.g. by mitochondria or by the NADPH oxidase Nox1, lipid peroxidation, enzymes of the energy metabolism, the deubiquitinase CYLD and the Bcl-2 family member Bmf have been suggested as further mediators of necroptosis [12]. In addition, our own group has previously identified the sphingolipid ceramide as a key effector of TNF-induced necroptosis [13,14]. Moreover, we have been able to show in a very recent study that, in contrast to previous assumptions [12], TNF-induced necroptosis is not mediated by the “PARP pathway” (a signaling cascade that involves overactivation of the DNA repair enzyme PARP-1, depletion of intracellular NAD^+^ and ATP, release of apoptosis-inducing factor from mitochondria, DNA fragmentation and cell death). Rather, necroptosis induced by TNF and the PARP pathway represent two independent and distinct routes to programmed necrosis [15].

In contrast to apoptosis, which depends essentially on the proteolytic activity of caspases, the role of proteolytic events for both regulation and execution of necroptosis/programmed necrosis is considerably less well characterized. Aside from a negative regulation of necroptosis by caspase-8 via cleavage and inactivation of RIPK1 [3], lysosomal proteases such as cathepsin B, D, calpains, granzymes and cys-cathepsins can substitute for caspases in some, but not all forms of programmed necrosis [16]. Also, the endoplasmic reticulum (ER) can induce programmed necrosis in response to cellular stress or uncontrolled release of calcium through calpain proteases [16,17]. Several groups (including our own) have independently observed that serine protease inhibitors such as tosyl phenylalanyl chloromethyl ketone (TPCK) can inhibit both necroptosis/programmed necrosis [18-21] and apoptosis [22]. For apoptosis, serine proteases have been found to complement or augment the function of caspases, e.g. granzyme B can stimulate apoptosis by cleavage of several procaspases, the pro-apoptotic protein Bid, or inhibitor of caspase-activated DNAse (ICAD) in cytotoxic T lymphocytes and natural killer cells [22]. For necroptosis/programmed necrosis, the identity of the relevant serine proteases and that of their substrates has remained largely obscure.

Here, we have identified the serine protease HtrA2/Omi as a key protease that mediates TNF-induced necroptosis. HtrA2/Omi is the mammalian homologue of the bacterial HtrA endoprotease and highly conserved from bacteria to mammalians. In the latter, HtrA2/Omi is involved in the degradation of misfolded proteins during conditions of cellular stress (e.g. ER stress, heat shock and ischemia/reperfusion) [23]. Deletion of HtrA2/Omi or mutations affecting its activity have been associated with neurodegeneration and Parkinson’s disease in mouse models [24] and patients [25]. In response to apoptotic stimuli, HtrA2/Omi is released from mitochondria into the cytoplasm, where it promotes apoptosis by binding and inhibiting IAP (inhibitor of apoptosis) proteins, thus releasing active caspases from their natural inhibitors. Independently, HtrA2/Omi degrades IAPs, the caspase-8 inhibitor Pea-15 and the anti-apoptotic protein HAX-1 through its serine protease activity, further promoting apoptosis [25].

In contrast to apoptosis, the molecular details of how HtrA2/Omi participates in necroptotic signaling are largely unknown. It has been reported that HtrA2/Omi can mediate caspase-independent PCD via its serine protease activity, e.g. upon interleukin-3 deprivation of the mouse pro-B cell line Ba/F3 [25], in imatinib-treated human leukemic cells [26], or in cytomegalovirus infection [27]. However, except for one study reporting cleavage and inactivation of RIPK1 by HtrA2/Omi [25], the substrates of HtrA2/Omi in necroptosis/programmed necrosis are unknown.

In the course of this study, we have identified ubiquitin C-terminal hydrolase (UCH-L1) as a second protease which participates in TNF-induced necroptosis downstream of HtrA2/Omi. UCH-L1 belongs to the family of cysteine proteases and functions as a deubiquitinase which generates, binds and stabilizes ubiquitin monomers, and thus can replenish the cellular monoubiquitin pool. Independently, UCH-L1 may act as an ubiquitin ligase [28], and may even have functions independent of the ubiquitin-proteasome system [29]. UCH-L1 is mainly expressed in neuronal tissues (very abundantly in the brain), in synovial membranes and in cells of the testis, ovaries, and kidney [28,30]. Abnormal expression of UCHL1 is found in many forms of cancer, including lung, colorectal, and pancreatic cancers, and may be related to tumor progression [29]. Aberrant expression of UCH-L1 has also been associated with neurodegenerative diseases, ischemic and traumatic brain injury [31]. Accordingly, and similar to HtrA2/Omi, mutations in UCH-L1 have been associated with Parkinson’s disease, as well as with other neurodegenerative disorders such as Alzheimer’s disease [30,32]. *De novo* expression of UCH-L1 is involved in podocyte injury and proteinuria in the kidney, possibly mediated through activation of the transcription factor NF-κB [30,31]. However, the true *in vivo* functions as well as the physiological substrates of UCHL1 remain unclear at present [29].

In this study, we have investigated the role of proteases in the regulation of TNF-induced necroptosis and establish two non-caspase proteases, the serine protease HtrA2/Omi and the deubiquitinase UCH-L1 as regulators of this form of PCD, simultaneously identifying two novel potential targets for therapeutic intervention.

## Results

### Inhibition of serine proteases, but not metalloproteases, cathepsin or calpain/cysteine proteases protects from TNF-induced necroptosis

**Figure 1:**
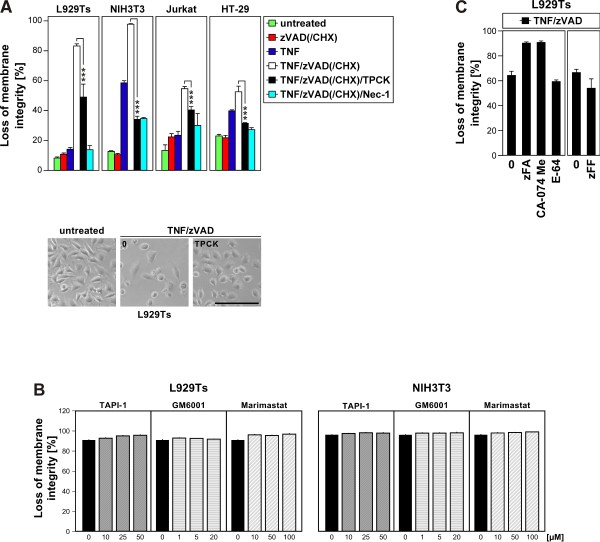
**Inhibition of serine proteases, but not metalloproteases, cathepsin or calpain/cysteine proteases protects from TNF-induced necroptosis. A**. Cells were stimulated or not with 100 ng/ml TNF for 5 (L929Ts), 16 (NIH3T3, HT-29), or 20 h (Jurkat) with optional addition of 20 (L929Ts, NIH3T3, HT-29) or 50 μM (Jurkat) of the broad-spectrum caspase inhibitor zVAD-fmk to prevent apoptosis, 2 (Jurkat) or 5 μg/ml (HT-29) cycloheximide (CHX) to sensitize for necroptosis [14] and 50 (L929Ts, NIH3T3) and 25 μM (Jurkat, HT-29) TPCK, or 50 μM of the necroptosis inhibitor necrostatin-1 (Nec-1, to confirm necroptosis). Subsequently, the cells were analyzed for loss of membrane integrity as a marker for cell death by PI staining and flow cytometry. Asterisks indicate statistical significance (t-test), *p < 0.05, **p < 0.01, ***p < 0.001. Micrographs show the morphology of untreated vs. necroptotic vs. L929Ts cells protected by TPCK. Scale bar: 100 μm. **B**. L929Ts and NIH3T3 cells were preincubated for 2 h with TAPI-1, GM 6001 and marimastat and subsequent addition of TNF/zVAD as in **A** before cell death was analyzed. **C**. L929Ts cells were incubated with TNF/zVAD as in **A** with optional addition of 20 μM zFA-fmk, CA-074 Me, E-64 or (in a separate experiment) zFF-fmk before cell death was analyzed. For all flow cytometric analyses of membrane integrity, we measured the percentage out of a total of 10,000 analyzed cells that show loss of membrane integrity (this is calculated as 100% minus the percentage of intact, large PI-negative cells to account for disintegrated dead cells that have lost their PI staining again due to diffusion). For all figures, representative data from one out of at least two or more experiments are shown and error bars indicate the standard deviations (SD) from at least triplicate determinations.

In a first set of experiments, we investigated the effects of different protease inhibitors on TNF-induced necroptosis. As shown in Figure 1A, TPCK, an inhibitor of chymotrypsin-like serine proteases significantly protected murine L929Ts fibrosarcoma cells (a tumor necrosis factor-related apoptosis-inducing ligand (TRAIL)-sensitive L929 subline derived in our laboratory [33]) from TNF-induced necroptosis, consistent with a previous study in parental L929 cells [21]. We found that TPKC also significantly diminished TNF-induced necroptosis in murine NIH3T3 fibroblasts cells as well as in human leukemic Jurkat T cells and in human HT-29 colorectal adenocarcinoma cells (Figure 1A) as further established cell systems for necroptosis [14,15,34]. We next investigated whether TNF-induced necroptosis is regulated by metalloproteinases. However, TAPI-1, an inhibitor of TACE (TNF-α converting enzyme, ADAM17) and other metalloproteinases, as well as GM 6001 and marimastat, two further broad-spectrum inhibitors of matrix metalloproteinases, had no inhibitory effect on TNF-induced necroptosis in L929Ts or NIH3T3 cells (Figure 1B). Likewise, inhibitors of the cysteine proteases cathepsin B/L (zFA-fmk), cathepsin B (Ca-074 Me), cathepsin L (zFF-fmk), as well as the broad-spectrum calpain/cysteine protease inhibitor E-64 did not protect L929Ts cells from TNF-induced necroptosis (Figure 1C), in line with previous findings [14,15,33]. In summary, these results suggest that chymotrypsin-like serine proteases participate in TNF-induced necroptosis in a cell type- and species-independent manner whereas inhibition of metalloproteinases, cathepsins and calpain/cysteine proteases has no major impact in this form of PCD.

### A screen for serine proteases relevant in TNF-induced necroptosis reveals HtrA2/Omi as a promising candidate

**Figure 2:**
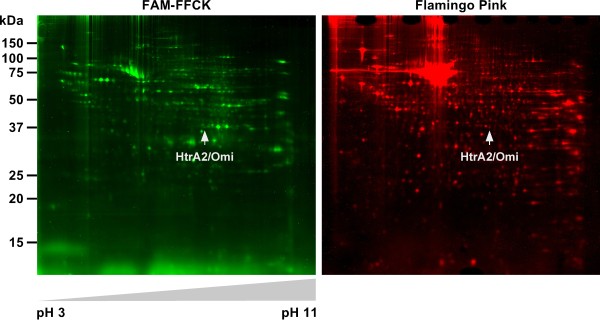
**Identification of HtrA2/Omi as a candidate serine protease involved in necroptosis.** L929Ts cells were stimulated with 100 ng/ml TNF for 5 h in combination with 20 μM zVAD-fmk, 2 μg/ml CHX (to enhance necroptosis) and 50 μM FAM-FFCK. Subsequently, lysates from the cells were separated by 2D gel electrophoresis and analyzed for protein spots labeled by FAM-FFCK (left panel, green), or for the total of all separated protein spots by staining with Flamingo Pink (right panel, red). The protein spot subsequently identified by mass spectrometry as HtrA2/Omi is indicated by arrows.

To identify the TPCK-sensitive serine protease(s) that regulate TNF-dependent necroptosis, we adapted an approach that had been previously employed to successfully identify proteases relevant for endoplasmic reticulum (ER) stress-induced caspase-independent PCD [35]. For this purpose, we induced necroptosis in L929Ts cells (to activate the relevant serine proteases) in the presence of a cell-permeable, active-site-directed, fluorescently labeled TPCK-derivative (FAM-FFCK), aiming to affinity-label only the subset of serine proteases that are activated during TNF-induced necroptosis. Lysates from the cells were separated by two-dimensional (2D) gel electrophoresis, and labeled protein spots were analyzed by mass spectrometry. Out of the analyzed 128 protein spots, 80 could be identified with high (“confirmed”) and 28 with lesser confidence (“candidate”). However, showing the limitations of this method and obviously due to a nonspecific background binding of FAM-FFCK, most of the 108 proteins turned out to be non-proteases. Nevertheless, the mitochondrial serine protease HtrA2/Omi was identified in this screen with high confidence (Figure 2), and we considered it as the most promising candidate, because it had been already associated with both caspase-dependent as well as caspase-independent PCD [25].

### HtrA2/Omi mediates TNF-induced necroptosis

**Figure 3:**
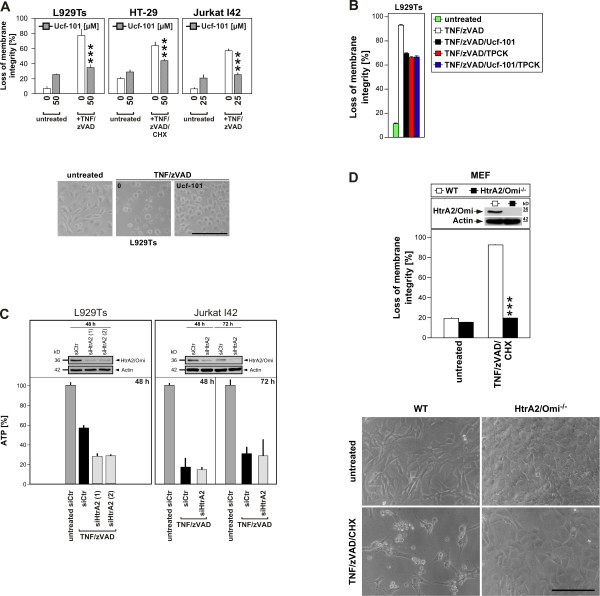
**HtrA2/Omi mediates TNF-induced necroptosis. A**. Cells were left without, or pretreated for 2 h with 25 (Jurkat I42) or 50 μM (L929Ts, HT-29) of Ucf-101. Subsequently, cells were further incubated for 5 (L929Ts), 16 (HT-29) or 6 h (Jurkat I42) without or with addition of 100 ng/ml TNF, 20 (L929Ts, HT-29) or 50 μM (Jurkat I42) zVAD-fmk and 5 μg/ml CHX (HT-29) before cell death was analyzed. ***p < 0.001. Micrographs show the morphology of untreated vs. necroptotic cells vs. L929Ts cells protected by Ucf-101. Scale bar: 100 μm. **B**. L929Ts cells were left untreated or treated with TNF/zVAD with or without Ucf-101 as in **A** with addition of 50 μM TPCK or not and analyzed as in **A**. **C**. Cells were transfected with siRNAs specific for murine (L929Ts) or human HtrA2/Omi (Jurkat I42), or a negative control siRNA (siCtr). After 48 or 72 h, cells were treated with 100 ng/ml TNF and 20 (L929Ts) or 50 μM (Jurkat I42) zVAD-fmk for another 5 (L929Ts) or 6 h (Jurkat I42) before the decrease of intracellular ATP levels was determined as a marker for cell death. Control Western blots of transfected but untreated cells were performed to verify downregulation of endogenous murine or human HtrA2/Omi. Detection of actin served as a loading control. **D**. Upper panel: wild-type (WT) and HtrA2/Omi-deficient MEF were stimulated with 100 ng/ml TNF, 20 μM zVAD-fmk and 1 μg/ml CHX for 16 h before cell death was determined. ***p < 0.001. Control Western blots show the presence or absence of murine HtrA2/Omi. Detection of actin served as a loading control. Lower panel: micrographs show the morphology of untreated and TNF/zVAD/CHX-treated WT vs. HtrA2/Omi-deficient MEF. Scale bar: 100 μm.

To investigate whether HtrA2/Omi was indeed functionally involved in the execution of TNF-induced necroptosis, we performed a first set of experiments in which we blocked the serine protease activity of HtrA2/Omi with the specific inhibitor Ucf-101 [36]. As shown in Figure 3A, treatment with Ucf-101 uniformly protected L929Ts, HT-29 and Jurkat I42 (a FADD-deficient, TNF-R2-transfected Jurkat subline which rapidly undergoes necroptosis in response to TNF [37]) cells from TNF-induced necroptosis, strongly suggesting that the serine protease activity of HtrA2/Omi is required for this process. Notably, incubation of L929Ts cells with Ucf-101 in combination with TPCK did not confer a stronger protection from necroptosis than the individual application of each inhibitor (Figure 3B), suggesting that both inhibitors do not act in an additive manner but rather via the same signaling pathway or even the same target (i.e. HtrA2/Omi). However, since results obtained with pharmacological inhibitors should be interpreted with a certain caution due to their potential nonspecific effects, we sought to further substantiate the function of HtrA2/Omi in TNF-induced necroptosis by selectively targeting its expression using RNA interference. As shown in Figure 3C, transfection of murine L929Ts or human Jurkat I42 cells with the corresponding siRNAs clearly downregulated the expression of HtrA2/Omi (although not completely). However, we did not detect a corresponding inhibition of TNF-induced necroptosis; i.e. loss of intracellular ATP measured as a marker for cell death was not prevented by HtrA2/Omi-specific siRNAs relative to a negative control siRNA (Figure 3C). As one possible explanation for this result, the achieved reduction of HtrA2/Omi expression (and thus activity) might not yet be sufficient to inhibit the death response. Alternatively, this result might indicate lack of a role for HtrA2/Omi in TNF-induced necroptosis and leave the possibility that cell death is mediated by TPCK-sensitive serine proteases other than HtrA2/Omi. Regardless of either interpretation, these results were not consistent with the data obtained by pharmacological inhibition with Ucf-101. To resolve this discrepancy, we obtained and analyzed mouse embryonic fibroblasts (MEF) from HtrA2/Omi-deficient mice in a direct genetic approach. As demonstrated previously [24], and as shown in Figure 3D, these cells are completely devoid of any residual HtrA2/Omi protein (and thus activity). In assays for TNF-induced necroptosis, HtrA2/Omi-deficient cells were fully protected (Figure 3D), confirming the results with Ucf-101 and in summary validating that HtrA2/Omi is a key mediator of TNF-induced necroptosis.

### HtrA2/Omi induces monoubiquitination rather than cleavage of its substrate UCH-L1 during TNF-induced necroptosis

**Figure 4:**
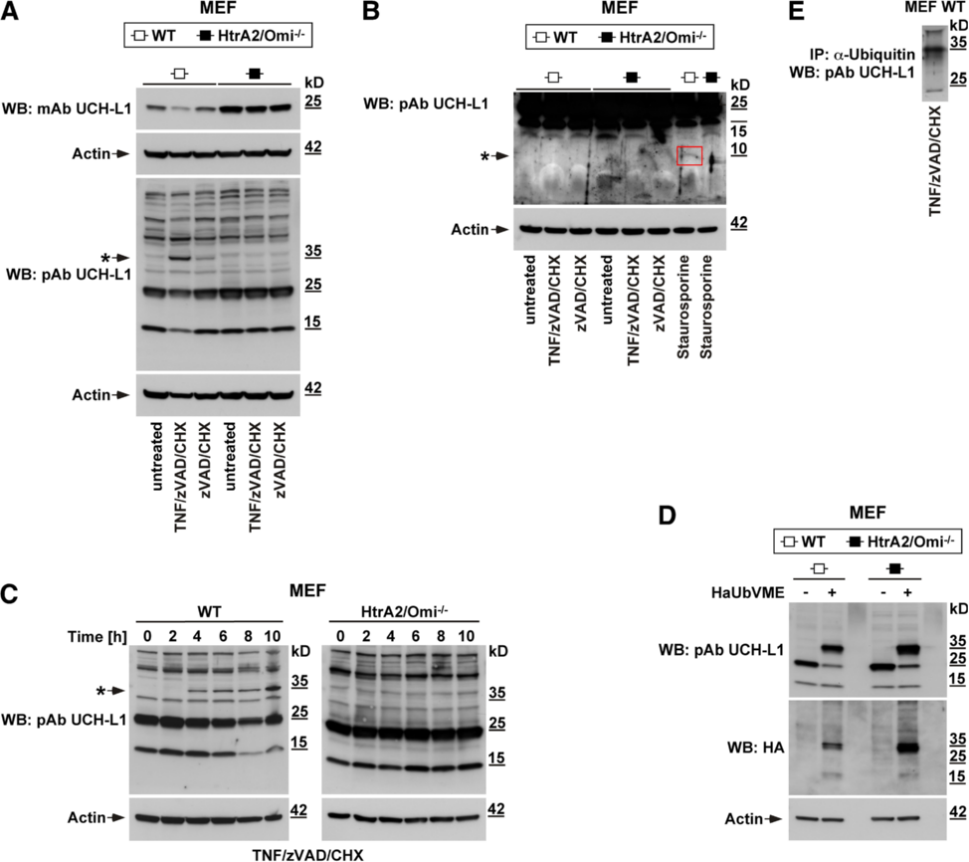
**HtrA2/Omi induces monoubiquitination rather than cleavage of its substrate UCH-L1 during TNF-induced necroptosis. A**. Wild-type (WT) and HtrA2/Omi-deficient MEF were left untreated or stimulated for 16 h with 20 μM zVAD-fmk and 1 μg/ml CHX in the presence (to induce necroptosis) or absence (for control) of 100 ng/ml TNF before UCH-L1 was detected with a monoclonal antibody that recognizes the full-length 25-kDa form of UCH-L1 (mAB UCH-L1) or, on a parallel blot, with a polyclonal antibody to detect all cleavage fragments of UCH-L1 (pAb UCH-L1). An asterisk marks the 35-kDa band corresponding to the predicted size of monoubiquitinated UCH-L1. **B**. WT and HtrA2/Omi-deficient MEF were stimulated as in **A**, and additionally with 0.5 μM staurosporine for 8 h. Lysates were separated on 10–20% w/v Tris-Tricine gels (Biorad) to resolve low molecular weight proteins and immunoblotted with pAb UCH-L1. The blot was deliberately overexposed to visualize faint cleavage fragments. The 10-kDa UCH-L1 cleavage fragment generated by HtrA2/Omi during staurosporine-induced apoptosis is indicated (arrow, red box). **C**. WT and HtrA2/Omi-deficient MEF were treated with TNF/zVAD/CHX as in **A** for the indicated times and analyzed for proteins reactive with pAb UCH-L1 by Western blot. An asterisk marks the appearance of the 35-kDa band identical to the predicted size of monoubiquitinated UCH-L1. **D**. Lysates from WT and HtrA2/Omi-deficient MEF were incubated with 20 μM of an HA-tagged ubiquitin-derived probe (HAUbVME) in 50 mM Tris, 150 mM NaCl, pH 8.0 for 90 min at 37°C and subsequently analyzed by immunoblotting with HA antibody and reanalyzed with pAB UCH-L1. In panels **A-D**, detection of actin served as a loading control. **E**. An immunoprecipitation was performed using lysates from necroptotic WT MEF (treated with TNF/zVAD/CHX as in **A**) and an antibody for ubiquitin. Subsequently, UCH-L1 was detected by Western blot using pAb UCH-L1.

The above results demonstrated that the protease activity of HtrA2/Omi is required for the necroptotic response to TNF, suggesting that necroptosis is relayed by proteolysis of HtrA2/Omi substrates. Since a previous study had shown that UCH-L1 is cleaved by HtrA2/Omi during staurosporine-induced apoptosis [38], we investigated whether UCH-L1 also served as a substrate and thus potential downstream effector of HtrA2/Omi in TNF-induced necroptosis. Initially supporting this assumption, Western blots revealed a decrease of the 25-kDa band representing full-length UCH-L in lysates from wild-type (WT) MEF after induction of necroptosis by TNF/zVAD/CHX (but not in untreated or zVAD/CHX-treated controls, Figure 4A). Moreover, this decrease was not detectable in HtrA2/Omi-deficient MEF (Figure 4A), and is therefore caused by HtrA2/Omi in the course of necroptosis. In addition, HtrA2/Omi-deficient MEF showed higher basal levels of UCH-L1 (Figure 4A), suggesting a constitutive negative impact of HtrA2/Omi on the levels of UCH-L1 in WT MEF. Since the monoclonal UCH-L1 antibody utilized in this experiment recognized only the full-length 25-kDa form of UCH-L1, we incubated a parallel blot with a polyclonal antibody for UCH-L1 to visualize additional cleavage fragments. As shown in Figure 4A, this antibody indeed detected a smaller band at 15 kDa. However, this band was uniformly present in WT and HtrA2/Omi-deficient MEF. Moreover, it did not increase but rather decreased upon induction of necroptosis in WT MEF (showing the same intensity pattern as full-length UCH-L1). Therefore, the 15-kDa band most likely represents a cleavage fragment of UCH-L1 which is constitutively generated by a protease distinct from HtrA2/Omi, and independent from necroptosis.

Park and colleagues have reported that HtrA2/Omi cleaves UCH-L1 during staurosporine-induced apoptosis, generating a 10-kDa cleavage fragment (although this was shown only *in vitro* and upon overexpression, but not for the endogenous proteins) [38]. We therefore included positive controls for cleavage of endogenous UCH-L1 by endogenous HtrA2/Omi by treating WT MEF with staurosporine, and additionally compared them to staurosporine-treated HtrA2/Omi-deficient MEF. Furthermore, we employed gel systems that specifically resolve low molecular weight fragments to detect any cleavage fragments that might have been missed in the experiment shown in Figure 4A. In line with the observations by Park and colleagues (but now shown for the first time for endogenous UCH-L1), we detected a very faint UCH-L1 cleavage fragment of 10 kDa in lysates from staurosporine-treated WT MEF. As an explanation for the low intensity of the 10-kDa fragment, Park and colleagues had previously been unable to detect endogenous cleavage fragments in WT MEF altogether (only *in vitro* and in overexpression systems), and had attributed this to an enhanced susceptibility of these fragments to degradation [38]. Nevertheless, the presence of this fragment in staurosporine-treated WT but not in HtrA2/Omi-deficient MEF (Figure 4B) confirmed that UCH-L1 is cleaved by HtrA2/Omi in staurosporine-induced apoptosis. In contrast, the 10-kDa fragment was clearly absent in all lysates from both WT and HtrA2/Omi-deficient MEF analyzed for TNF-induced necroptosis as well as the accompanying controls (Figure 4B). Given these results, we considered it unlikely that the observed decrease of the 25-kDa full-length UCH-L1 band in necroptotic WT MEF was resulting from a direct proteolytic cleavage of UCH-L1 by HtrA2/Omi.

Searching for an alternative explanation, we noticed that the disappearance of the 25-kDa UCH-L1 band during TNF-induced necroptosis was accompanied by the concurrent appearance of a prominent band of ~35 kDa (Figure 4A). Like the 25-kDa band, this band was completely absent in HtrA2/Omi-deficient as well as in untreated WT MEF (and only very faintly detectable as a background band in control WT MEF treated with zVAD/CHX). To obtain further insight, we extended the above analysis in a timecourse experiment. As shown in Figure 4C, induction of necroptosis in WT MEF by TNF/zVAD/CHX caused the appearance of the ~35-kDa band within 4 h of treatment and again reduced the levels of the 25-kDa UCH-L1 form (most clearly visible after 8 h). Again, this was not detectable in HtrA2/Omi-deficient MEF (Figure 4C), in line with the results shown in Figure 4A, and once more demonstrating that these changes are mediated by HtrA2/Omi. Interestingly, a band of ~35 kDa reactive with UCH-L1 antibodies has also been described by other groups, and has been suggested to represent a monoubiquitinated form of UCH-L1 [29,32,39]. To clarify whether this was the case, we incubated lysates from WT and HtrA2/Omi-deficient MEF with an ubiquitin-derived probe tagged to hemagglutinin (HA) that covalently binds to deubiquitinating enzymes such as UCH-L1 [32]. In Western blots for UCH-L1, incubation of the lysates with this probe caused a shift of the full-length UCH-L1 band from 25 kDa to ~35 kDa. Moreover, an antibody against the HA tag of the probe selectively reacted with this ~35-kDa band (Figure 4D). We additionally immunoprecipitated ubiquitinated proteins from WT MEF after induction of necroptosis with TNF/zVAD/CHX and performed Western blots for UCH-L1, again detecting a band at ~35 kDa (Figure 4E). In summary, these results confirm that the size shift from 25 kDa to ~35 kDa is indeed caused by monoubiquitination of UCH-L1. It is noteworthy that two of the above groups have independently shown that this modification leads to activation of UCH-L1 [29,32], prompting us to investigate the functional relevance of UCH-L1 activity for TNF-mediated necroptosis in the next set of experiments.

### Inhibition of UCH-L1 protects from TNF-induced necroptosis

**Figure 5:**
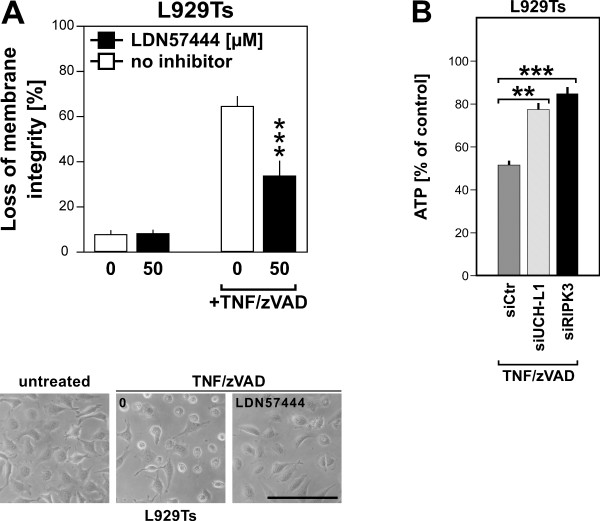
**Inhibition of UCH-L1 protects from TNF-induced necroptosis. A**. L929Ts cells were prestimulated for 3 h with 50 μM of the UCH-L1 inhibitor LDN57444 or left unstimulated before addition of 100 ng/ml TNF and 20 μM zVAD-fmk for 5 h. Subsequently, cell death was analyzed by PI staining and flow cytometry. Asterisks indicate statistical significance (t-test), ***p < 0.001. Micrographs additionally show the morphology of untreated L929Ts cells vs. necroptotic cells vs. cells protected by LDN57444. Scale bar: 100 μm. **B**. L929Ts cells were transfected with an siRNA that does not elicit an RNAi response (negative control, siCtr), with an siRNA specific for murine UCH-L1, and with an siRNA specific for murine RIPK3 (positive control for protection from necroptosis, siRIPK3) as described in “Methods.” 48 h after transfection, cells were treated with 100 ng/ml TNF and 20 μM zVAD-fmk for another 5 h before the decrease of intracellular ATP levels was determined as a marker for cell death. ATP levels are shown relative to controls that were not treated with TNF/zVAD. Asterisks indicate statistical significance (t-test), **p < 0.01, ***p < 0.001.

For this purpose, we employed LDN57444, a previously described active site-directed inhibitor which specifically targets the enzymatic activity of UCH-L1 [40]. As shown in Figure 5A, treatment of L929Ts cells with LDN57444 significantly protected from TNF-mediated necroptosis. To exclude that this was due to nonspecific effects of this pharmacological inhibitor, we additionally downregulated UCH-L1 by RNA interference, measuring loss of intracellular ATP as a marker for TNF/zVAD-induced necroptosis. Compared to L929Ts cells transfected with a negative control siRNA, transfection with an siRNA specific for UCH-L1 significantly inhibited loss of ATP, almost as effective as transfection with an siRNA specific for RIPK3, which we used as a positive control to validate the assay (Figure 5B). In summary, the above results support the hypothesis that UCH-L1 is not cleaved by, but rather indirectly activated downstream of HtrA2/Omi, further relaying the necroptotic signals elicited by TNF.

### UCH-L1 is a mediator of caspase-independent, non-apoptotic cell death in diseased kidney podocytes

Remarkably, UCH-L1 has also been associated with increased cell death in patients with kidney failure. In particular, *de novo* expression and thus increased UCH-L1 activity in kidney podocytes was found in specific, mostly irreversible forms of glomerular injury in patients, rats and mice and is apparently responsible for disease aggravation in experimental models of membranous nephropathy [30,31]. Accordingly, inhibition of UCH-L1 with LDN57444 diminished kidney damage in these models whereas overexpression of UCH-L1 enhanced podocyte destruction. At present, it is however completely unclear whether death of podocytes in response to increased UCH-L1 activity is mediated by apoptosis, by autophagic mechanisms, by necroptosis or other forms of programmed necrosis. For apoptosis, evidence for podocyte death is scarce, suggesting that apoptosis is not a general pathway of podocyte loss *in vivo*[41]. As a second potential mode of PCD, autophagy has rather been associated with a healthy and differentiated status of podocytes, implicating that podocyte autophagy is a protective instead of pro-death pathway in glomerular disease [41]. Finally, necroptosis in podocytes has been investigated so far in only one study, where healthy podocytes (which do not express UCH-L1 [28]) proved resistant to both necroptosis and apoptosis [42].

**Figure 6:**
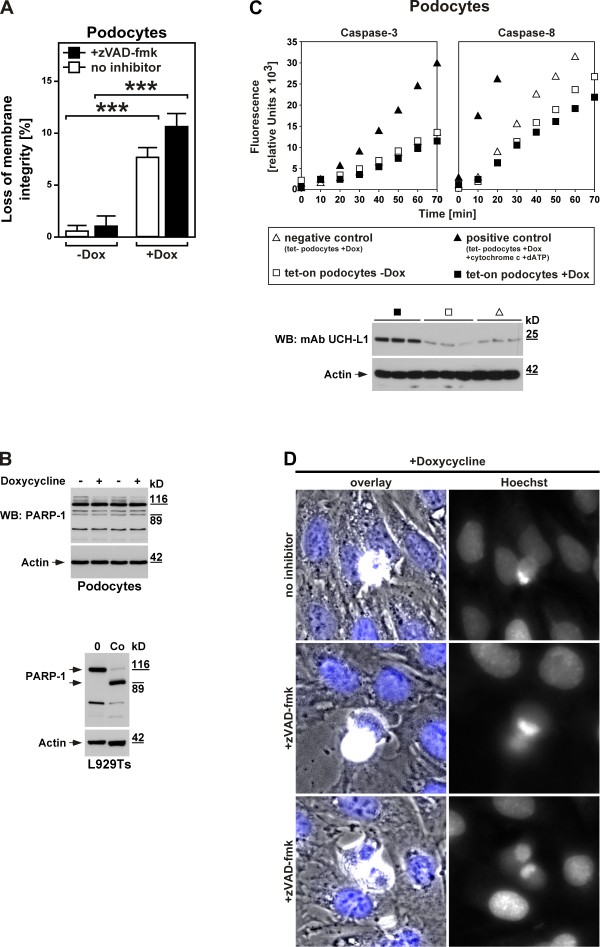
**UCH-L1 as a mediator of caspase-independent, non-apoptotic cell death in diseased kidney podocytes. A**. UCH-L1 tet-on podocytes were treated with 20 ng/ml doxycycline for 72 hours (+Dox) or not (-Dox) and 50 μM zVAD-fmk or no inhibitor. Cell death was measured by trypan blue staining. ***p < 0.001. **B**. UCH-L1 tet-on podocytes were left untreated (-) or treated with doxycycline as in **A** (+) before PARP-1-reactive bands were detected by immunoblotting. Cell lysates from untreated and apoptotic L929Ts cells (Co, treated with 100 ng/ml TNF and 2 μg/ml CHX for 1 h) are shown as controls. Full-length and cleaved PARP-1 is marked by arrows. Detection of actin served as a loading control. **C**. Aliquots from the stimulations in **B** were also analyzed for caspase activity. As negative control, tet- podocytes were treated with doxycycline as in **A**; as positive control, lysates from doxycyline-treated tet- podocytes were incubated with cytochrome c and dATP to activate caspases. Subsequently, the activity of caspases-3 and -8 was determined by measuring the cleavage of fluorogenic substrates (zIETD-afc and zDEVD-afc) over 70 minutes. The Western blot below shows that UCH-L1 is indeed upregulated in doxycycline-treated but not untreated UCH-L1 tet-on podocytes and also not in doxycycline-treated negative control tet- podocytes. Treatment with doxycycline was performed as in **A**. UCH-L1 was detected with mAb UCH-L1, detection of actin served as a loading control. **D**. Cell death was induced in UCH-L1 tet-on podocytes by treatment with doxycycline as in **A** in the presence of 50 μM zVAD-fmk (+zVAD-fmk, middle and lower micrographs) or no inhibitor (upper micrographs). Micrographs show the morphology of dying cells within a monolayer of healthy cells (overlay, nuclei are stained blue), and in parallel the nuclear morphology of the same cells after staining with Hoechst dye (Hoechst). Original magnification: x 400.

To explore the mode of cell death that podocytes undergo in response to an increase in UCH-L1 expression/activity, we utilized murine podocytes stably transduced with a doxycycline-inducible overexpression construct for UCH-L1 (UCH-L1 tet-on podocytes). In a first approach, we investigated cell death in untreated and doxycycline-treated UCH-L1 tet-on podocytes directly. As shown in Figure 6A, cell death in untreated UCH-L1 tet-on podocytes was negligible whereas induction of UCH-L1 expression by doxycycline significantly increased the numbers of dying podocytes (thereby also demonstrating the functionality of the system). More importantly, the addition of zVAD-fmk as a broad-spectrum inhibitor of caspases and thus of apoptosis did not inhibit but rather enhanced UCH-L1-dependent cell death. We and others have previously observed this effect of zVAD-fmk in necroptosis [14,33,43], excluding that *de novo* expression and thus increased UCH-L1 activity causes death of podocytes by apoptosis but rather pointing to programmed necrosis/necroptosis as the responsible suicide program.

To extend these results, we investigated cleavage of PARP-1, a DNA-associating repair enzyme which is inactivated in apoptosis by caspase-3-dependent processing of the mature 116-kDa protein to an 89-kDa cleavage product [44]. When we analyzed lysates from UCH-L1 tet-on podocytes treated with doxycycline for 72 h or not in Western blots, the full-length 116-kDa PARP-1 band was uniformly visible in all samples, together with a pattern of additional bands. However, this pattern did not change upon treatment with doxycycline (Figure 6B). In particular, the characteristic disappearance of the full-length 116-kDa PARP-1 band as well as the corresponding increase of the 89-kDa cleavage fragment that we have previously observed for apoptosis in multiple studies [13,15,33], and which is also shown for control in L929Ts cells (Figure 6B) could not be detected. Given that caspase-3 acts downstream of all other apoptotic caspases as the central effector caspase of both extrinsic and intrinsic apoptosis, these results provided a second line of evidence that caspase activation and thus apoptosis seems not to occur during UCH-L1-mediated death of kidney podocytes.

To address this point in more detail, we directly measured the activity of caspase-3 and caspase-8 (as the major initiator caspase activated by death receptors). As shown in Figure 6C, no increase in caspase-3 or caspase-8 activity beyond the already present basal levels was detectable in doxycycline-treated (i.e. UCH-L1-overexpressing, Western Blot in Figure 6C) vs. untreated UCH-L1 tet-on podocytes or vs. negative controls (doxycycline-treated podocytes that are stably transduced with empty vector; tet- podocytes). In contrast, the activity of both caspases was clearly increased in positive control lysates from doxycycline-treated tet- podocytes treated with cytochrome c and dATP to validate the assay, in summary further corroborating the assumption that UCH-L1-mediated death of podocytes occurs without activation of caspases and thus in a non-apoptotic manner.

Finally, when analyzed by microscopy, doxycycline-treated UCH-L1 tet-on podocytes did not display typical apoptotic changes such as membrane blebbing, type 2 chromatin condensation and accumulation of fragmented chromatin at the nuclear periphery which we had earlier observed for apoptosis in other cell systems [33]. Rather, only an incomplete, lumpy condensation of chromatin was detectable that has previously been associated with programmed necrosis/necroptosis rather than apoptosis [16]. Moreover, and as shown above for cell death, the addition of zVAD-fmk did not affect the changes in the cellular and nuclear morphology of podocytes caused by doxycycline-induced overexpression of UCH-L1 (Figure 6D). Altogether, these results rule out caspase-dependent apoptosis but rather favor caspase-independent, non-apoptotic forms of cell death such as programmed necrosis or necroptosis as the most probable cause for UCH-L1-mediated podocyte death.

### Inhibition of UCH-L1 protects podocytes from TNF-induced necroptosis

**Figure 7:**
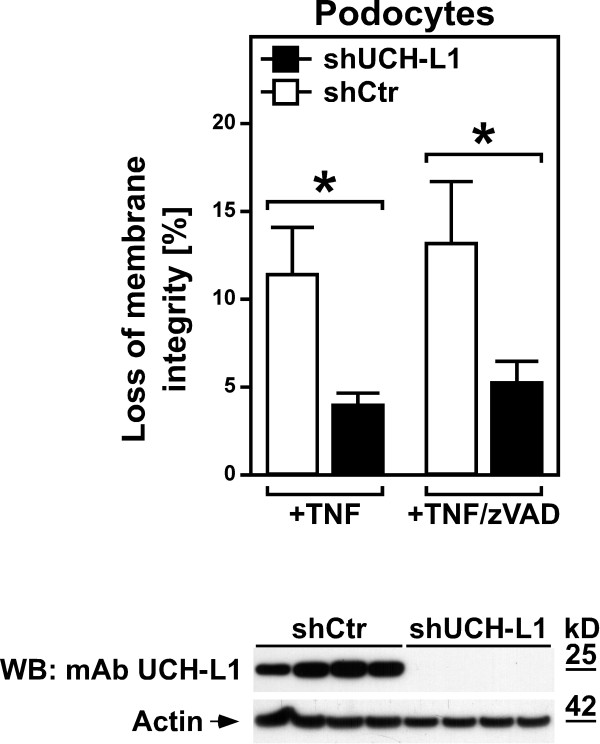
**Inhibition of UCH-L1 protects podocytes from TNF-induced necroptosis.** Podocytes stably transfected with an shRNA construct that causes permanent knockdown of UCH-L1 (shUCH-L1) or with a scrambled negative control shRNA (shCtr) were treated with 100 ng/ml TNF in the presence of 50 μM zVAD-fmk or vehicle for 3 h before loss of membrane integrity as a marker for cell death was measured by trypan blue staining. Asterisks indicate statistical significance (t-test), *p < 0.05. The Western blot below was performed to demonstrate the permanent knockdown of UCH-L1 in shUCH-L1 podocytes, but not in shCtr podocytes. UCH-L1 was detected with mAb UCH-L1, detection of actin served as a loading control. For each stable transfectant, lysates from four independent flasks were analyzed.

As a central proinflammatory cytokine, TNF may also contribute to inflammatory reactions in the kidney and thus to subsequent podocyte injury. We therefore wanted to determine whether UCH-L1 can act as a mediator of TNF-induced necroptosis not only in L929Ts cells (as shown in Figure 5), but also in podocytes. For this purpose, we analyzed podocytes stably transfected with an shRNA construct that causes permanent knockdown of UCH-L1 or with a scrambled negative control shRNA [30]. As shown in Figure 7, podocytes with stable downregulation of UCH-L1 were significantly protected from TNF-induced cell death when compared to control podocytes. Moreover, and identical to podocyte death caused by UCH-L1 overexpression (Figure 6A), the addition of zVAD-fmk did not prevent TNF-induced cell death, demonstrating that TNF indeed elicits necroptosis in podocytes, and that UCH-L1 represents a downstream mediator of the necroptotic signaling cascade of TNF also in podocytes.

## Discussion

The impact of caspase-independent, non-apoptotic PCD such as necroptosis/programmed necrosis has become increasingly clear in the last years. This is particularly true for pathological processes, for example renal [42], cardiac and retinal ischemia/reperfusion injury, hyperacute shock [45], brain damage or pancreatitis [12], Huntington’s, Parkinson’s and Alzheimer’s disease, epilepsy, muscular dystrophy, as well as for the destruction of cells by pathogens such as vaccinia virus, HIV, Shigella and Salmonella [2,12,46,47]. The option to therapeutically interfere with necroptosis/programmed necrosis has raised great expectations [12]. In consequence, a better knowledge of the still incompletely understood signaling pathways and the associated components will facilitate future strategies to interfere with damage induced by necroptosis/programmed necrosis (e.g. in shock, stroke, myocardial infarction or kidney failure). Here, we have identified the proteases HtrA2/Omi and UCH-L1 as two such components of TNF-induced necroptosis, and thus revealed two novel targets for therapeutic intervention, e.g. by future Ucf-101- or LDN57444-derived drugs suited for use in patients.

**Figure 8:**
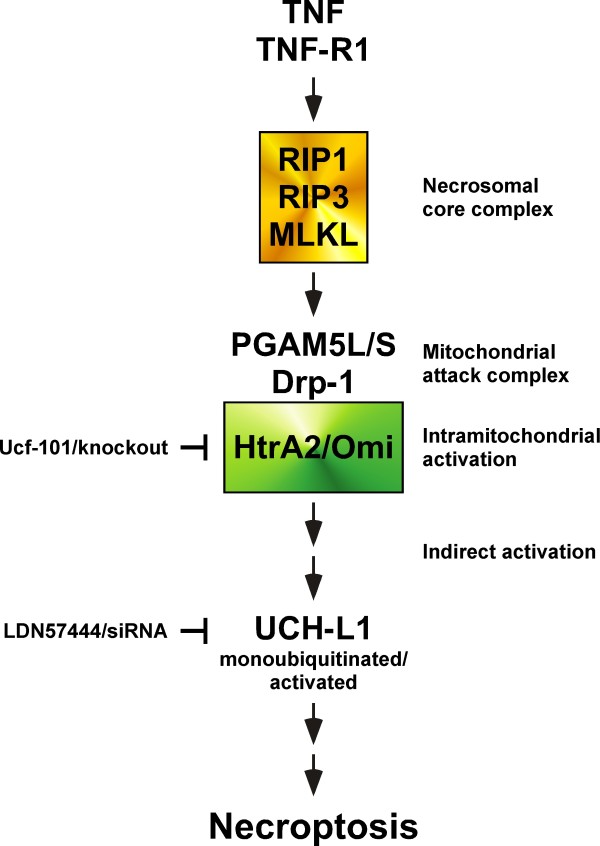
**HtrA2/Omi and UCH-L1 as novel components of TNF-induced necroptosis.** The scheme depicts the proposed roles of HtrA2/Omi and UCH-L1 in TNF-induced necroptosis. Binding of TNF to TNF-R1 triggers activation of the necrosomal core complex consisting of RIPK1, RIPK3 and MLKL. Subsequently, the proteins PGAM5L/S and Drp-1 form the mitochondrial attack complex, resulting in the intramitochondrial activation of HtrA2/Omi. Activated HtrA2/Omi then (by cleavage of yet unidentified intramitochondrial substrates) indirectly causes monoubiquitination and activation of UCH-L1, and finally, necroptosis. Accordingly, inhibition of HtrA2/Omi (Ucf-101, knockout) or UCH-L1 (LDN57444, siRNA) protects from necroptosis. Please see Discussion for further details.

Based upon the results of our study, we propose the model shown in Figure 8 to integrate HtrA2/Omi and UCH-L1 into the known signaling pathways of TNF-induced necroptosis. In this model, binding of TNF to TNF-R1 induces activation of the kinases RIPK1, RIPK3, and of MLKL as components of the necrosomal core complex. Notably, we have been unable to detect HtrA2/Omi as part of the necroptotic TNF-R1 signaling complex in preliminary experiments (D. A. and S. S., unpublished observations), and no other study has yet reported an association of HtrA2/Omi with components of the TNF-R1 signaling complex during necroptosis. This is also consistent with reports showing that, in contrast to apoptosis, HtrA2/Omi is not released from mitochondria during TNF-induced necroptosis [23,48]. In summary, these findings argue against a direct interaction of HtrA2/Omi with RIPK1, RIPK3 or MLKL but instead suggest that HtrA2/Omi is activated indirectly within the mitochondria. As the most likely mechanism, MLKL has been found to activate the phosphatases PGAM5L/S, which in turn couple to the mitochondrial protein Drp-1, and as a mitochondrial attack complex [11], may cause the subsequent intramitochondrial activation of HtrA2/Omi. Consistent with a function of HtrA2/Omi in TNF-induced necroptosis despite this intramitochondrial localization, inhibition of HtrA2/Omi activity by Ucf-101 or by genetic deletion (knockout) blocks the necroptotic signal of TNF (this was similarly observed for Ucf-101 in an independent study in neutrophils, where the authors also concluded that HtrA2/Omi mediates necroptosis through its serine protease properties from *within* the mitochondria [48]). Downstream of HtrA2/Omi, our data identify UCH-L1 as another, novel component of the signaling cascade. In contrast to staurosporine-induced apoptosis, where HtrA2/Omi translocates into the cytosol and directly cleaves and thus inactivates UCH-L1 [38], the intramitochondrial localization of HtrA2/Omi during TNF-induced necroptosis prevents a direct interaction of both proteins. Rather, and also explaining why we did not see a direct cleavage (and thus inactivation) of UCH-L1 by HtrA2/Omi, HtrA2/Omi seems to act indirectly, by yet unknown mechanism (e.g. cleavage of unidentified intramitochondrial substrates), causing the monoubiquitination and activation of UCH-L1, finally resulting in necroptosis (which can accordingly be blocked via LDN57444 or by RNA interference).

As a side note, UCH-L1 belongs to the family of cysteine proteases, and we wondered why the broad-spectrum calpain/cysteine protease inhibitor E-64 did not confer any significant protection from TNF-induced necroptosis in the experiments performed in this study (Figure 1C) or in additional control experiments (D. A., J. S. and S. V., unpublished observations). To the best of our knowledge, inhibition of UCH-L1 by E-64 has also not been shown in any other study. As a possible explanation, UCH-L1 is an “atypical” cysteine protease because its active site is misaligned when compared to productive cysteine proteases [29]. Therefore, a general cysteine protease inhibitor such as E-64 may have only limited impact on the activity of UCH-L1, in contrast to a specific inhibitor such as LDN57444 or to inhibition of UCH-L1 by RNA interference (which clearly protected L929Ts cells or podocytes from TNF-induced necroptosis, Figure 5A-B, Figure 7).

We would also like to point out that HtrA2/Omi and UCH-L1 obviously represent important, but most certainly not the only factors transmitting the necroptotic death signals of TNF downstream of RIPK1/RIPK3/MLKL. Whereas HtrA2/Omi is expressed ubiquitously [23], the expression of UCH-L1 is restricted to certain tissues [28,30]. Therefore, in tissues that do not express UCH-L1, necroptosis must be relayed by additional, independent factors. Notably, the brain is an organ where a rapid and efficient apoptotic elimination of cells is dangerous, and where alternative, caspase-independent forms of PCD predominate [16]. The brain is also the organ with the highest expression of UCH-L1 in the entire body [32], suggesting that a deregulation of UCH-L1 activity in the brain may contribute to necroptotic damage, e.g. after traumatic injury [31] or after stroke (i.e. ischemia/reperfusion damage). Interestingly, both UCH-L1 as well as HtrA2/Omi have been associated with Parkinson’s disease, although a connection to necroptosis has not been investigated so far. Moreover, recent studies have found that necroptosis is also the predominant damage mechanism in ischemia/reperfusion damage in the kidney [42,49], in summary indicating that both brain and kidney are organs where therapeutic strategies aiming to interfere with the necroptotic actions of HtrA2/Omi and UCH-L1 may be worthwhile options to consider for the future, e.g. with regard to stroke or kidney failure.

## Conclusions

We have identified the proteases HtrA2/Omi and UCH-L1 as two crucial components of TNF-induced necroptosis, and thus provided evidence that proteolysis is not only critical for the regulation and execution of apoptosis, but also essential for caspase-independent forms of PCD. A model that integrates HtrA2/Omi and UCH-L1 into the known signaling cascades of TNF-mediated necroptosis is shown in Figure 8. With HtrA2/Omi and UCH-L1, we have also revealed two novel targets for therapeutic intervention, which may assist in developing strategies for the treatment of damage induced by necroptosis/programmed necrosis (e.g. in organs such as the kidney and the brain, caused by stroke or kidney failure).

## Methods

### Reagents

Highly purified human recombinant TNF was provided by BASF Bioresearch (Ludwigshafen, Germany). Benzyloxycarbonyl-Val-Ala-Asp(OMe)-fluoromethylketone (zVAD) was from Bachem (Bubendorf, Switzerland). TPCK, marimastat, benzyloxycarbonyl-Phe-Ala-fluoromethylketone (zFA-fmk) and trans-Epoxysuccinyl-L-leucylamido(4-guanidino)butane (E-64), were purchased from Sigma (Deisenhofen, Germany), necrostatin-1, TAPI-1, GM6001, 5-[5-(2-nitrophenyl)furfurylidine]-1,3-diphenyl-2-thiobarbituric acid (Ucf-101), benzyloxycarbonyl-Phe-Phe-fluoromethylketone (zFF-fmk) and LDN57444 from Merck Millipore (Darmstadt, Germany), and N-[L-3-trans-(propylcarbamoyl)-oxirane-2-carbonyl]-L-Ile-L-Pro methyl ester (CA-074 Me) from Biomol (Hamburg, Germany). Carboxyfluorescein-labeled phenylalanine chloromethyl ketone (FAM-FFCK) was from Immuno Chemistry Technologies (Bloomington, MN, USA). Staurosporine was obtained from Selleckchem (Munich, Germany), Ubiquitin vinyl methyl ester, HA-tag (HaUbVME) from Enzo Life Sciences (Lausen, Switzerland).

### Cell culture

L929Ts is a TRAIL-sensitive L929 subline derived in our laboratory [33]. NIH3T3 cells naturally expressing RIPK3 and therefore sensitive to necroptosis have been previously described [15,50,51]. Jurkat and HT-29 cells were obtained from the American Type Culture Collection (ATCC, Manassas, VA, USA). Jurkat I42 cells were a kind gift from Francis Ka-Ming Chan (Worcester, MA, USA). Immortalized MEF deficient for HtrA2/Omi [24] and their WT counterparts were originally generated by Julian Downward (London, U. K.) and kindly provided by Thomas Langer (Cologne, Germany). Cells were cultivated in DMEM (NIH3T3, MEF), or a mixture of Click’s/RPMI 1640 medium (all other cell lines) supplemented with 10% v/v fetal calf serum and 2 mM glutamine at 37°C in a humidified incubator containing 5% w/v CO_2_. Media were additionally supplemented with 1 mM sodium pyruvate (HT-29) and 50 μg/ml each of streptomycin and penicillin. Murine podocytes (a kind gift from K. H. Endlich, Greifswald) were cultured as described [52]. For differentiation, podocytes were cultured for 14 days under non-permissive conditions (37°C, 7.4% w/v CO_2_, RPMI 1640 supplemented with 10% v/v fetal calf serum, 10 mM N-2-hydroxyethylpiperazine-N0-2-ethanesulfonic acid, 1 mM sodium pyruvate, 100 U/ml penicillin, 100 mg/ml streptomycin).

### Flow cytometric analysis of membrane integrity

Cells were seeded in twelve-well plates at 5 x 10^4^ cells/well. Following treatment, both detached and adherent cells were collected by centrifugation. The cells were resuspended in PBS/5 mM EDTA containing 2 μg/ml propidium iodide (PI), and the red fluorescence was measured on a FACSCalibur flow cytometer (Becton Dickinson).

### Statistical analysis

*p* values were calculated using Student’s t-test. Statistical significance is denoted by *p < 0.05, **p < 0.01, ***p < 0.001.

### Microscopy

For documentation of cell morphology, images from unfixed cells were obtained using an Axiovert 10 microscope (Zeiss, Oberkochen, Germany) and a DS-5M-L1 digital sight camera system (Nikon, Düsseldorf, Germany).

### 2D gel electrophoresis, image analysis and spot picking

The two-dimensional gel electrophoresis was essentially performed as described before [53]. After harvesting, cells were lysed on ice for 10 min in TNE buffer (50 mM Tris pH 8.0, 1% v/v NP40, 2 mM EDTA) containing 10 μg/ml protease inhibitor cocktail (Roche, Mannheim, Germany). For protein precipitation, trichloroacetic acid (TCA) was added to the protein lysate to a final concentration of 10% v/v . The mixture was incubated for 30 min on ice and centrifuged at 10,000 × g at 4°C for 20 min. The supernatant was removed, ice-cold acetone was added to wash the pellet and the sample was centrifuged as above. After removal of the supernatant, the pellet was air dried and resuspended in lysis buffer (pH 8.5) containing 7 M urea, 2 M thiourea, 30 mM Tris, 4% w/v CHAPS. The supernatant containing the solubilized proteins was recovered after centrifugation for 20 min at 20,000 × g at 4°C. A total amount of 250 μg of protein was mixed with rehydration buffer (7 M urea, 2 M thiourea, 4% w/v CHAPS, 2% v/v immobilized pH gradient (IPG) buffer pH 3–11 and 2% w/v DTT) and applied by cup-loading onto 24 cm non-linear pH 3–11 IPG gel strips for isoelectric focusing (IEF). The second dimension was performed on 26 × 20 cm large 12.5% w/v gels after reduction and alkylation using the Ettan DALTsix large vertical electrophoresis system from GE Healthcare (Munich, Germany). The gels were removed from the glass plates, mounted on a non-backed gel frame, and scanned on a Typhoon Trio imager (GE Healthcare) at green fluorescence. Subsequently, the gels were stained overnight with Flamingo Pink (Bio-Rad, Munich, Germany), and scanned again at red fluorescence. The obtained images were analyzed using Image Master 6.0 (GE Healthcare). Selected spots were picked with a 2 mm picking head. The picked gels were again scanned to verify the correct location of the punched spots.

### In-gel tryptic digestion and mass spectrometry

The punched gel spots were sequentially washed with water, with 50 mM ammonium bicarbonate (ABC) in 50% v/v MeOH, with 70% v/v acetonitrile (ACN), and dehydrated in pure ACN. ACN was evaporated in a SpeedVac centrifuge (ThermoFisher Scientific, Dreieich, Germany), and the dry gel pieces were subjected to in-gel digestion with 100 ng porcine sequencing-grade trypsin (Serva, Heidelberg, Germany) in 25 mM ABC at 37°C overnight. For peptide extraction, 20 μl of 0.1% v/v trifluoroacetic acid (TFA) in ACN was added and the samples were sonicated for 15 min. The supernatants were removed and the gel spots were again incubated with 20 μl of 0.1% v/v TFA in ACN for 10 min. The supernatants of both steps were combined, dried in a SpeedVac centrifuge, redissolved in 0.8 μl MALDI matrix solution (3.2 mg/ml α-cyanohydroxycinnamic acid (Sigma) in 65% v/v ACN/0.1% v/v TFA), spotted onto 384-well stainless steel MALDI plates and air-dried. Spectra were acquired on an AB SCIEX MALDI-TOF/TOF 5800 (AB SCIEX, Darmstadt, Germany) mass spectrometer in positive ion mode. For MS measurements, 2000 shots were accumulated in the mass range of 800–4000 m/z. Default calibration was performed using the 4700 Proteomics Analyzer Standards Kit, while MS measurements were calibrated internally using trypsin and contaminant peaks (842.509, 2211.105, 2225.120 and 2807.315 Da). Precursor selection for MS/MS analysis was achieved using the 4000 Series Explorer Software (AB SCIEX) with acquisition of the 20 most intense precursors (S/N > 20), beginning with the strongest first. All MS/MS spectra were acquired with 1 KV collision energy at ambient air (CID medium: 1.25 x 10–6 Torr) using 3000 laser shots. For peptide identification, MALDI-TOF/TOF MS/MS raw files were searched using ABSciex GPS software (Version 3.6, build 332) with the following pre-filter settings: only peaks within a mass range from 60 Da to the precursor mass minus 35 Da and S/N ratio above 10 were used. Spectra were searched with Mascot (version 2.2.04, Matrix Science, London, U.K) against the Swissprot database using Mus musculus as a taxonomy filter (15 Feb 2011, 16345 sequences) and the following parameters: precursor tolerance, 50 ppm; MSMS tol, 0.3 Da; max missed cleavages 2. Oxidation (M) was set as a variable modification, while carbamidiomethylation (C) was set as a fixed modification. Proteins were considered identified when either 2 peptides were identified with a confidence interval ≥ 99% (*p* < 0.01) or 3 peptides ≥ 95% (*p* < 0.05).

### RNA interference

The validated siRNA specific for human HtrA2/Omi (ID # s654), the predesigned siRNAs specific for murine HtrA2/Omi (ID # s82292, s82292) murine UCH-L1 (ID # s75710), murine RIPK3 (ID # s80755) as well as the negative control siRNA (ID # AM4611) were obtained from Life Technologies, Darmstadt, Germany. L929Ts cells were transfected with 150 pmol siRNA by Amaxa nucleofection (Lonza, Cologne, Germany), using solution V and program T-20. Jurkat I42 cells were transfected with 30 pmol siRNA and HiPerFect transfection reagent (Qiagen, Hilden, Germany).

### Measurement of intracellular ATP levels

The intracellular ATP content of cells was determined with the Cell Titer Glo Assay Kit (Promega, Mannheim, Germany) following the instructions of the manufacturer.

### Immunoblots

Unless otherwise indicated, cells were harvested after treatment and lysed at 4°C in TNE buffer containing 150 mM NaCl, 10 μg/ml protease inhibitor cocktail, 1 mM sodium orthovanadate and 5 mM NaF. Identical amounts of cell protein per lane were resolved by electrophoresis on SDS polyacrylamide gels. After electrophoretic transfer to nitrocellulose, reactive proteins were detected using antisera specific for actin (sc-1615, Santa Cruz, Heidelberg, Germany; A1978, Sigma), HtrA2/Omi (ab32092, Abcam, Cambridge, UK), UCH-L1 (rat monoclonal, clone U104, generated as outlined below, or rabbit polyclonal, CL95101, Cedarlane, Burlington, Ontario, Canada), HA (1187423, Roche), PARP-1 (9542, Cell Signaling, Danvers, MA, USA) and the ECL detection kit (GE Healthcare). Equal loading as well as efficiency of transfer was routinely verified for all Western blots by Ponceau S staining, and by reprobing the membranes for actin.

### Generation of monoclonal UCH-L1 antibodies

Wistar rats were initially immunized intraperitoneally (i.p.) with 100 μg of purified UCH-L1 (kindly provided by Gregory A. Petsko, Waltham, MA, USA) in 60 μl phosphate buffer saline (PBS) emulsified with 40 μl of Gerbu adjuvant MM (Gerbu Biotechnik, Heidelberg, Germany). The rats were boosted i.p. on days 14 and 21 with 50 μg of purified protein emulsified with 20% v/v of the adjuvant. The last two doses (50 μg UCH-L1 in PBS) were administered on days 28 and 29 without adjuvant, while the fusion was done on day 30. Spleen cells from immunized animals were collected and fused with Ag8.653 myeloma cells using polyethylene glycol 1500 (Roche). The fused cells were cultured in selection medium (HAT, Sigma) for 10 days and screened by ELISA for anti-UCH-L1 antibodies. Hybridoma clones producing anti-UCH-L1 monoclonal antibodies (mAbs) were then cultivated in serum-free medium and the mAbs were purified using protein G affinity chromatography (GE Healthcare). The isotype of the anti-UCH-L (U104) clone (IgG1, λ) was determined by using ELISA rat mAb isotyping kit (ThermoFisher).

### Immunoprecipitations

Cellular lysates were precleared with GammaBind G-sepharose (GE Healthcare) and immunoprecipitation was performed over night on ice using anti-ubiquitin IgG1 monoclonal antibody (MAB1510, Merck Millipore, 1:100 dilution). After collection of the immunecomplexes with GammaBind G-sepharose and three washing steps in lysis buffer, the immunoprecipitated proteins were analyzed by SDS-PAGE and Western blot.

### Generation of stably transfected podocytes with inducible overexpression or downregulation of UCH-L1

For inducible overexpression of UCH-L1, the Retro-X Tet-On Advanced Inducible Expression System (Clontech, Mountain View, CA, USA) was used according to the manufacturers’ instructions. Briefly, wildtype murine UCH-L1 was amplified by polymerase chain reaction from murine podocytes using the following primers: mUCHL1-pRetro-fw 5′CTAGGCGGCCGCGCCACCATGCAGCTGAAGCCGATGGA′3; mUCHL1-pRetro-rev 5′CTAGACGCGTTTAAGCTGCTTTGCAGAGAG′3 and subsequently cloned into the multiple cloning site of the pRetroX-Tight-Pur vector using NotI and MluI (ThermoFisher). The sequence of UCH-L1 was verified by sequencing (Eurofins MWG Operon, Ebersberg, Germany). For virus production, phoenix ecotropic packaging cells were transfected using DNA/CaCl_2_ precipitation with the pRetroX-Tet-On Advanced vector, with the pRetroX-Tight-Pur-UCH-L1 vector or the pRetroX-TightPur empty vector as a control, respectively. The virus-containing supernatant of the pRetroX-Tet-On transfected phoenix cells was transferred to a 10 cm plate containing podocyte target cells at around 50% to 60% confluence; the infection steps were repeated twice. Selection for integration of the pRetroX-Tet-On Advanced expression plasmid was performed with G418 (500 μg/ml, Life Technologies) for 7 days. Afterwards, the virus-containing supernatant of the pRetroX-Tight-Pur-UCH-L1 transfected phoenix cells was transferred to the pRetroX-Tet-On Advanced transduced podocyte target cells; the infection steps were again repeated twice. Selection for integration of the pRetroX-Tight-Pur-UCH-L1 plasmid was performed with puromycin (1.5 μg/ml, Sigma). For negative control experiments, the pRetroX-Tight-Pur vector was transduced without insert (tet-) into the pRetroX-Tet-On Advanced expressing podocytes. For induction of UCH-L1 overexpression, UCH-L1 tet-on or tet- podocytes were cultured in the presence of tetracycline free medium (PAN-Biotech, Aidenbach, Germany) supplemented with 20 ng/ml doxycycline or without doxycycline for control. For stable knockdown experiments, shRNA627 to murine UCH-L1 or scrambled shRNA for control was overexpressed in podocytes as described before [30].

### Analysis of caspase activity, cell death, and cellular and nuclear morphology in podocytes

10^5^ differentiated UCH-L1 tet-on or tet- podocytes were plated in 6-well plates in tetracycline-free RPMI 1640 medium (Life Technologies) supplemented with 10% v/v fetal calf serum, 10 mM N-2-hydroxyethylpiperazine-N0-2-ethanesulfonic acid, 1 mM sodium pyruvate, 100 U/ml penicillin and 100 mg/ml streptomycin. UCH-L1 overexpression was induced with 20 ng/ml doxycycline for 72 hours or not. For measurements of caspase activity, cells were collected and lysed in a buffer containing 10 mM Hepes pH 7.4, 142 mM KCl, 5 mM MgCl_2_, 1 mM EGTA, 0.2% v/v NP40, 1 mM DTT and 2 mM Pefabloc (Roche). To generate positive controls, 20 μg of cells lysate were equilibrated for 1 h at 30°C after the addition of 1 mM dATP and 10 μM cytochrome c to permit activation of caspases. Subsequently, 100 μl of caspase buffer (20 mM Pipes, 100 mM NaCl, 10 mM DTT, 1 mM EDTA, 0.1% w/v CHAPS, 10% w/v sucrose, pH 7.2) containing 100 μM zDEVD-afc (benzyloxycarbonyl-Asp(OMe)-Glu(OMe)-Val-DL-Asp(OMe)-7-aminotrifluoromethylcoumarin, Merck Millipore) or zIETD-afc benzyloxycarbonyl-Ile-Glu(OMe)-Thr-DL-Asp(OMe)-7-aminotrifluoromethylcoumarin (Merck Millipore) were added to 10 μl of cytosolic extract (10 μg protein) and incubated at 37°C. The release of afc was measured as emission at 505 nm upon excitation at 405 nm using an Infinite M200 fluorimeter equipped with a thermostated plate reader (Tecan, Crailsheim, Germany). For measurements of podocyte death, viable and dead cells were detached with trypsin and counted in a Neubauer chamber after 0.1% w/v trypan blue (Life Technologies) staining. The percentage of dead cells was calculated and plotted as mean +/- SEM, n = 12 per condition. To analyze cellular and nuclear morphology, cells were stained with Hoechst dye (10 μg/ml, Life Technologies) for 5 min and DNA condensation in UCH-L1 tet-on podocytes with or without induced UCH-L1 overexpression for 72 hours was evaluated under an Axio Observer A1 microscope (Zeiss) using the axiovision software (Zeiss).

### Analysis of TNF-induced cell death in podocytes

Differentiated sh627 and scrambled shRNA control podocytes were plated at a density of 10^4^ cells per 6-well plate. After 48 hours, cells were treated with 100 ng/ml murine TNF (PeproTech, Hamburg, Germany) with addition of 50 μM zVAD-fmk or vehicle (ethanol) as control for 3 hours. Cells were detached with trypsin and the amount of dead and living cells was counted in a Neubauer chamber following staining with 0.1% w/v trypan blue. The percentage of dead cells was calculated and plotted as mean +/- SEM, n = 12 per condition.

## Competing interests

The authors declare that they have no competing interests.

## Authors’ contributions

JS, SV, DK, OJ, CMS, SS and DA designed research; JS, SV, SM, AT and CMS performed research; JS, SV, DK, AT, OJ, CMS, SS and DA analyzed data, DA wrote the manuscript. All authors read and approved the final manuscript.
